# Clinical application of PARP inhibitors in ovarian cancer: from molecular mechanisms to the current status

**DOI:** 10.1186/s13048-023-01094-5

**Published:** 2023-01-07

**Authors:** Yongsong Wu, Shilin Xu, Shanshan Cheng, Jiani Yang, Yu Wang

**Affiliations:** 1 Department of Shanghai Key Laboratory of Maternal Fetal Medicine, Shanghai First Maternity and Infant Hospital, School of Medicine, Tongji University, Shanghai200092, China; 2 Obstetrics and Gynecology, School of Medicine, Renji Hospital, Shanghai Jiaotong University, Shanghai, China

**Keywords:** PARP inhibitor, Ovarian cancer, BRCA, Synthetic lethality, Homologous recombination repair, Chemotherapy resistance, Biomarkers, Side effect

## Abstract

As a kind of gynecological tumor, ovarian cancer is not as common as cervical cancer and breast cancer, but its malignant degree is higher. Despite the increasingly mature treatment of ovarian cancer, the five-year survival rate of patients is still less than 50%. Based on the concept of synthetic lethality, poly (ADP- ribose) polymerase (PARP) inhibitors target tumor cells with defects in homologous recombination repair(HRR), the most significant being the target gene Breast cancer susceptibility genes(BRCA). PARP inhibitors capture PARP-1 protein at the site of DNA damage to destroy the original reaction, causing the accumulation of PARP-DNA nucleoprotein complexes, resulting in DNA double-strand breaks(DSBs) and cell death. PARP inhibitors have been approved for the treatment of ovarian cancer for several years and achieved good results. However, with the widespread use of PARP inhibitors, more and more attention has been paid to drug resistance and side effects. Therefore, further research is needed to understand the mechanism of PARP inhibitors, to be familiar with the adverse reactions of the drug, to explore the markers of its efficacy and prognosis, and to deal with its drug resistance. This review elaborates the use of PARP inhibitors in ovarian cancer.

## Introduction

As a common gynecological tumor, ovarian cancer has many different tissue types. 90% of them are epithelial cell type, and the rest are non-epithelial ovarian cancers. There are also some rare pathological types such as small cell carcinoma and carcinosarcoma. Different tissue types of ovarian cancer have different molecular changes, clinical behaviors and therapeutic effects. However, the prognosis of ovarian cancer in different tissues is generally dismal, in which rare carcinosarcoma has both epithelial and sarcomatous components, and the prognosis is very poor [1]. In industrialized countries, ovarian cancer is the tumor with the highest number of deaths among cervical cancer, uterine cancer, and other gynecological tumors [2]. According to a statistical forecast in the United States, it is estimated that more than 20, 000 people will suffer from ovarian cancer and more than 13,000 will die of ovarian cancer in 2021. Its morbidity ranks outside the top 10 among American women, but it ranks fifth in mortality [3]. One of the reasons is that ovarian cancer is mostly occult onset, it was in its late stage when the definite diagnosis came out. The standard of treatment for advanced ovarian cancer is surgery combined with chemotherapy. In recent years, neoadjuvant chemotherapy (NACT) before surgery is also an option. But at present, there is a lack of consensus on who is the most suitable for this strategy and how to choose a specific scheme. Importantly, it can initially predict the sensitivity of patients to chemotherapy and the risk of recurrence [4]. Even so, the risk of recurrence is high and the prognosis is poor. Therefore, PARP inhibitors selectively targeting tumor cells that cannot repair DNA double-strand breaks have been developed, and have achieved significant clinical efficacy in the treatment of recurrent ovarian cancer [5].

It is known that BRCA1 and BRCA2 are tumor suppressor genes involved in DNA repair, which are closely related to the incidence of ovarian cancer. BRCA1/2 germline mutation is the strongest known genetic risk factor for epithelial ovarian cancer and are found in 6–15% of women with epithelial ovarian cancer. The status of BRCA1/2 can be used for counseling patients with expected survival because BRCA1/2 carriers with epithelial ovarian cancer respond better to platinum-based chemotherapy than non-carriers. This leads to higher survival rates, although the disease is usually diagnosed at a later stage and at a higher level [6]. People with BRCA gene mutations have an increased risk of breast cancer and ovarian cancer at any age [7]. Furthermore, germline or somatic aberrations in the DNA damage repair genes are found in 19% of primary prostate cancer and nearly 23% of metastatic castration-resistant prostate cancer, which impaired the integrity of the genome. Therefore, several PARP inhibitors have been studied in patients with mCRPC and are effective against germline BRCA2 mutants [8]. The concept of synthetic lethality is indispensable to understand the mechanism of PARP inhibitors and their relationship with BRCA genes. As early as 2005, the concept has been proposed by Bryant and Farmer et al., that is, the change of a single gene/protein does not affect the survival of cells, but the simultaneous change of two or more genes/proteins is fatal to cells [9, 10]. Based on this concept, PARP inhibitors target tumor cells with BRCA gene mutations without affecting the survival of normal cells. In the past few years, the clinical use of PARP inhibitors has greatly increased and achieved successful results.

This review mainly introduces the concepts of homologous recombination and synthetic lethality, analyzes how PARP inhibitors make use of this mechanism, as well as a general analysis of drug resistance, enumerates several tumor markers that predict the prognosis and therapeutic effect of ovarian cancer, summarizes the application status and achievements of PARP inhibitors in patients with ovarian cancer, lists the potential problems and prospects of PARP inhibitors(PRAPis).

## Homologous recombination and synthetic lethality

The survival and proliferation of normal cells are inseparable from the genetic code. Human DNA is constantly exposed to DNA damage agents, so DNA damage repair is the core of cell survival. Cells have their own precise and interrelated DNA repair system [11]. The lack of this system will lead to gene mutation, which will affect the biological function and proliferation of normal cells, and even lead to carcinogenesis.

The damage to DNA can be divided into DNA single-strand break (SSB) and double-strand break, among which single-strand break is the most common. Four of the six DNA repair pathways found to target single strand mutations are base excision repair (BER), nucleotide excision repair (NER), mismatch repair (MMR) and translocation synthesis [12, 13]. In the case of SSB repair defects or DNA double strand breaks, cells can also deal with such situations through recombination repair. Recombination repair includes homologous recombination repair (HRR) and nonhomologous end-jointing (NHEJ). Compared with SSB, there is no intact complementary chain that acts as a template for DSB, so homologous recombination repair replicates the original DNA sequence through its sister chromatid as template, so the newborn DNA sequence has high fidelity. While nonhomologous end-jointing, as the name implies, the two segments of DNA can connect to each other without homology, so there may be some differences between the newborn DNA sequence and the original sequence [14–16]. The occurrence of ovarian cancer and breast cancer is related to the mutation of BRCA gene involved in DNA double strand repair [7].

**Fig. 1:**
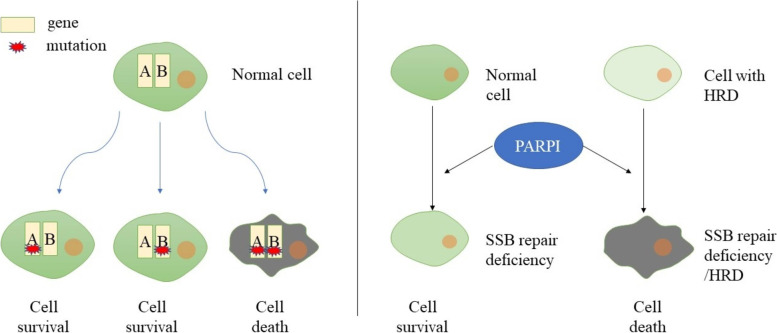
[The concept of synthetic lethality and its application in ovarian cancer] Synthetic lethality is defined as a combination of mutations in two or more separate genes that lead to cell death. For example, if a cell suffers the mutation of either gene A or B alone, it can still survive, while mutation of both gene A and B will lead to cell death

In recent years, the concept of synthetic lethality has aroused great interest among scientists. If any mutation or dysfunction between the two genes does not affect the survival of the cell, but when they are mutated or dysfunctional at the same time, the effect on the cell is fatal, there is synthetical lethality between the two genes [17, 18](Fig. 1). It is well known that genetic mutations in tumor cells are very common. BRCA gene mutation in ovarian cancer is a good starting point. It is known that homologous recombination repair involved in BRCA protein can be used as a way to remedy the defect of SSB repair function. Since BRCA gene mutation in ovarian cancer is involved in the occurrence of cancer, if a substance blocks the cell’s SSB repair pathway, it can completely destroy the cells' gene repair function without affecting normal cells, so the substance can mainly attack tumor cells and less affect normal cells [19]. This requires us to understand the general process of SSB repair.

## The function of PARP and PARP inhibitor

**Fig. 2:**
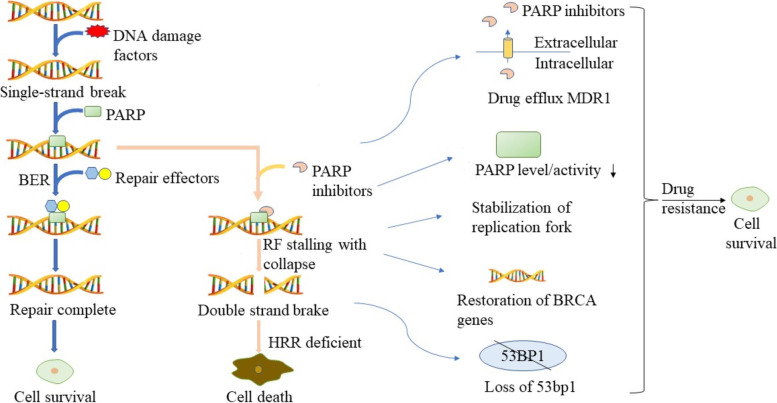
[The action and resistance mechanism of PARP inhibitors] The different panels of the figure show the action and resistance mechanisms of PARP inhibitors, respectively

As a ribozyme, Poly (ADP-ribose) (PAR) polymerase-1 (PARP-1) is one of the main roles in SSB repair, and the most studied and major role in the PARP family is PARP1 [20]. PARP can sense DNA gaps, then transfer single ADP-ribose or PAR to itself or other proteins, and then recruit DNA repair proteins and other proteins to DNA damage sites to help DNA repair [21, 22]. PARP1 has DNA binding and unbinding states. The non-binding state can sense DNA damage and bind to DNA SSB through the zinc finger structure, and use NAD as the substrate to catalyze the transfer of ADP-ribose or PAR to itself and other receptor proteins to form a PARP chain. Subsequently, PARP1 recruited various DNA repair effectors, such as deoxyribonucleic acid polymerase β and deoxyribonucleic acid ligase III, and molecular scaffold protein XRCC1, which can branch PARP chain polymers to DNA damage sites to assist in DNA repair [23, 24]. After the completion of the repair, PARP1 experienced molecular changes that led to the decrease of DNA affinity, which was finally released from the repaired DNA and returned to the original DNA unbound state [25](Fig. 2).

The killing effect of PARP inhibitors on cancer cells is not only to inhibit the function of PARP, but also has other mechanisms. The most classical mechanism is the trapping effect of PARP inhibitors on PARP [26]. After binding to the NAD binding pocket of PARP, PARP inhibitors not only affect the role of PARP proteins, but also fix PARP proteins on damaged DNA without dissociation, resulting in the accumulation of PARP-DNA nuclear protein complex and destruction of replication forks. In the end, not only SSB cannot be repaired, RF stalling leads to degradation of the highly cytotoxic DSBs [27]. Therefore, PARP inhibitors can lead to a single DNA break not only can't be repaired, but also developed into the DSB. For ovarian cancer cells with HRR deficiency, the accumulation of highly cytotoxic DSB will eventually lead to cell death, while cells with normal HRR repair pathway can repair DSB. Therefore, PARP inhibitors can target cancer cells with mutations in the BRCA gene.

As mentioned above, in addition to HRR, NHEJ is also a compensatory repair way for DSB. It has been reported that the synthetic lethal form is also related to the inhibition of NHEJ. Unlike HRR, NHEJ is faster than homologous recombination, and recent evidence suggests that it plays a role in the entire cell cycle, not just in the G1 phase. Except the known proteins such as Ku70/80, DNA-PKcs, Artemis, DNA ligase IV-XRCC4, DNA pol λ/μ, and XLF, New proteins have been found to be associated with NHEJ, namely PAXX, MRI/CYREN, IFFO1, ERCC6L2, TARDBP of TDP-43, and RNase H2. Among them, MRI/CYREN has dual functions. It stimulates NHEJ in G1 phase of the cell cycle, while it inhibits the pathway in S phase and G2 phase [28].

PARP-1 inhibits NHEJ by PARylating Ku70/Ku80 and the catalytic subunit of DNA-PKcs [29]. PARP inhibitors block this inhibition and reinforce the more error-prone repair pathway, causing more mutations in cells, and resulting in cell death [30]. At present, it is not clear the extent of the lethality of these mechanisms in cancer cells. Therefore, it is necessary to provide personalized targeted therapy for gene mutations in different subgroups of patients.

## PARP inhibitor resistance

With the increase in the clinical use of PARP inhibitors, resistance has attracted increasing attention. Through the current study, we have learned that in HR-deficient (HRD) tumors, PARP inhibitors can capture PARP1, resulting in DNA damage repair disorder and resulting in cell death through synthetic lethality [29]. At present, we know that DSB is repaired through the unstable NHEJ repair pathway especially when HR repairs defects, and the two repair pathways one goes against each other [31]. Therefore, any factors that promote the recovery of HR repair function or lead to the inhibition of the NHEJ repair pathway may cause PARP inhibitor resistance. In addition, the number and functional site of PARP inhibitors also affect the sensitivity of drug action to a certain extent.

### The HR function recovery

From what we have talked about, we know that function reversing will lead to PARP inhibitors resistance. Therefore, some changes in epigenetic modification of the HR repair protein influence the PARP inhibitors resistance. It has been found that the deletion of promoter methylation of the BRCA1 gene is sufficient to restore HR and lead to resistance [32]. Pingping Fang et al. reported that transcriptional factor FOXM1 can cause the overexpression of genes associated with the HR repairing pathway in cancer cells, related to olaparib resistance [33]. When patients took olaparib, the FOXM1 pathway is activated and BRCA1 and RAD51 express, acquiring partial resistance. Thiobacillomycin can reduce the expression of FOXM1 and increase the expression of some apoptosis-related genes. It also helps PARP inhibitors to capture PARP1 [33]. USP15 promotes the retention of BARD1/BRCA1 which prevents the damaging terminal from resection. It is reported by Peng et al. that, overexpression of USP15 caused the resistance of PARP inhibitors [34]. Molecular chaperone protein, HSP90, has the function of resisting protein folding wrongly by BRCA mutations. Then it forms unstable structures and causes protein degradation. HSP90 stabilizes mBRCA protein and helps RAD51 loading into RF to promote DNA damage repair [35].

### Inhibition of the NHEJ repair pathway

The NHEJ repair pathway is more unstable and sometimes produces more repairing errors while the HR repair pathway is more reliable. And the NHEJ will result in the accumulation of damaged DNA and finally cause apoptosis. The functions of the NHEJ repair pathway and the HR pathway have been traded off. Therefore, when the repair function of NHEJ is inhibited, part of the DNA damage can only pass through HR repair, which also leads to resistance to PARP inhibitors.

#### Deletion of 53BP1

Base excision at the damaged end of DNA plays an important role in repair. Relevant studies found that protein 53BP1 further prevented the aggravation of DNA damage by recruiting REV7-SHLD1-SHLD2-SHLD3(Shieldin) complex and blocking the resection of bases, resulting in DNA damage not passing through HR repair but starting the NHEJ repair [36]. The NHEJ repair pathway results in the accumulation of damaged DNA and causes apoptosis.

#### Enhanced DNA damage repair function

In addition to changes in HR repair and NHEJ repair function, any factors that enhance DNA damage repair (DDR) ability may lead to drug resistance. Pingping Fang et al. reported that FOXM1 up-regulated the expression of genes related to the DDR repair pathway in olaparib-less sensitive cancer cells [33]. In the study by Giovannini et al., reducing the repair efficiency of oxidative damage result in resistance to PARPi in BRCA1 mutation-deficient cells. Oxidative damage produces some abnormal bases including Go (8-oxyguanidine). Specific DNA glycosylation enzymes such as OGG1 and MYH can remove Go and repair DNA damage through the BER pathway. This study also found that siRNA-mediated RNA knockout of OGG1 or MYH affected SSB repair, resulting in reversion of HR repair and resistance to PARP inhibitors [37]. Bellio et al. found that PARP inhibitor can affect the increase of CD133^+^ and CD117^+^ ovarian cancer stem cell populations (CSC). The prolonged cell cycle of these stem cell populations after PARP inhibitors’ treatment may be related to enhanced DNA damage repair. In addition, there may be a potential mechanism for enhancing DDR capacity in the CSC population, as more abundant RAD51 and DMC1 lesions were observed [38]. Euchromatic histone-lysine N-methyltransferases 1 and 2 (EHMT1/2) catalyze dimethylation of histone H3 lysine 9 (H3K9me2) leading to epigenetic silencing [39]. EHMT1/2 complex can recruit some factors involved in HR and non-homologous terminal linking (NHEJ), including BRCA1, to increase DNA damage repair [40]. Watson et al. observed that increased expression of H3K9me2 and EHMT1/2 in BRCA2 mutation-resistant ovarian cancer patients could also support this [41]. Liu et al. demonstrated that drug resistance in epithelial ovarian cancer (EOC) cells may be associated with increased aldehyde dehydrogenase (ALDH) activity, possibly because PARP inhibitors induce increased BRD4 expression. Enhanced ALDH1A1 terminal junction activity with microhomology mediation was observed in EOC cells inactivated with BRCA2, which is associated with HR repair recovery [42].

### Changes in the mode of drug action

#### Change of actional target

While PARP1 is an important member of PARP family and has something to do with repairing DNA damage sites. After PARylation of the PAR chain, PARP1 can recruit some DNA-damage repair factors to help realize its function [43]. The PARylation of PARG counteracts and prevents HR from occurring. PARP family members work primarily by binding to PARP1 [44]. Therefore, any factor that affects the function of PARP1 will lead to failure or resistance of PARP inhibitors.

#### PARP1 decreases

The reduced number of PARP results in a lack of PARP inhibitor targets, leading to resistance. Current studies have observed primary resistance to PARP inhibitors in cells with low PARP1 expression [45]. In addition, Gogola et al. observed PARP inhibitor resistance in tumor cells by introducing two shRNA-mediated PARG loss, which is more common in BRCA2 mutated tumors. Therefore, the endogenous activity of PAR sugar hydrolase(PARG) has a certain influence on the action of PARP inhibitors. When RAPRi does not completely block the accumulation of PARP, the loss of PARG activity causes downstream proteins of PARP1 to continue to perform DNA damage repair functions, possibly leading to resistance [46].

#### PARP1 domain mutation

IN addition to changes in the number of PARP, any factors affecting PARP inhibitors binding to PARP may be related to resistance. Mutations in the DNA-bound zinc finger (ZnF) domain of PARP1 lead to abnormal capture of PARP inhibitors, which affects its function and leads to drug resistance [47].

### Decreased drug concentration

P-glycoprotein (P-gp) is a drug transporter that is related to the resistance in many drugs by reducing transmembrane transportation and this reduces the intracellular concentration of drugs [48]. Therefore it reduces the drug effect. In some primary ovarian tumors, mRNA of P-gp has been detected. The higher it expresses, the worse overall survival will be observed. Nowadays, most PARP inhibitors are multi-drug resistance protein 1(MDR1) substrates, and over-expression of MDR1 causes resistance.

### The stabilization of RF

PARP1 and BRCA2 can stabilize the replication fork (RF), thus preventing the degradation of RF by MRE11 [49, 50]. The factors affecting MRE11 can affect the resistance of PARP inhibitors through RF protection. FANCD2 effector protein is associated with DNA replication and damage repair. Kais et al. found that MRE11 mediated RF degradation was inhibited by overexpression of FANCD2 [51]. Mani et al. Found that Hedgehog (Hh) / Glioma associated protein 1 (GLI1) was abnormally activated in some (Ovarian carcinosarcoma)OCS and affected the function of RF by regulating the transcription of FANCD2, thereby promoting DNA damage repair. Inducing the down-regulation of Hh / GLI1 can lead to HR repair deficiency, so it is related to improving resistance [52]. In addition, studies have found that mir-493-5p expressed in BRCA2 mutant cancer cells affects the stability of single-strand annealing (SSA), R-loops, and replication fork, which may lead to PARP inhibitor resistance. The expression of mir-493-5p significantly down-regulates MRE11 and results in RF protection [53].

## Biomarkers related to PARP inhibitors

With the increasing application of PARP inhibitors, people gradually began to look for some effective biomarkers to accurately predict the response of PARP inhibitors and monitor the effect during the treatment. So as to predict the target population and avoid resistance. It is meaningful in making personal treatments for patients. This is in line with the developing trend of precision medicine.

### Myriad genetics BRACAnalysis CDx platform

We now know that PARP inhibitors take effect in BRCAness tumors that are defective in homologous recombination through synthetic lethality. Therefore, any biomarker that can detect BRCA mutation is a key indicator for the clinical prediction of PARP inhibitors [54, 55]. The FDA approved myriad mychoice ® CDX (myriad genetic laboratories, Inc.) to be used in diagnosing genomic scars caused by DNA damage including telomeric allelic imbalance (TAI), loss of heterozygosity (LOH) and large-scale transition (LST). The test assesses and quantifies HRD status by measuring genomic scarring. The test-positive patients whose score is over 42 may be beneficial from PARP inhibitors. Besides, it is helpful in assessing the prognosis and long-term response of PARPi [56].

### Sensitive to platinum

Platinum drugs have been used as first-line chemotherapy for (Epithelial ovarian cancer)EOC, and platinum sensitivity can also predict the status of HRD in tumor cells to a certain extent. The NOVA trial demonstrated that platinum-sensitive patients may benefit from PARPi [56, 57]. In the clinical, platinum sensitivity is also a relatively convenient and low-cost indicator to predict the effectiveness of PARP inhibitors, but it is not absolute. Some patients are sensitive to platinum but not sensitive to PARP inhibitors; conversely, there are also patients with obvious benefits from PARP inhibitors who are resistant to platinum. In a phase IB study, advanced ovarian cancer patients who were platinum-resistant could respond partly to PARP inhibitors [58]. This suggests that although the molecular pathways of the two drugs are partially overlapping, but not completely. More details about the mechanisms still need further exploration.

### Biomarkers associated with homologous recombination repair

A. RAD51 HR proficient cancers can form RAD51 foci which is a critical step in the HR pathway and its test is predictive of PARP inhibitors. RAD51 is a key factor in the synthesis of new DNA templates during DNA damage repair, which can be loaded into the site of DNA damage to form nucleoprotein filaments. Castroviejo-Bermejo et al. used formalin-fixed samples demonstrating that the RAD51 assay can identify HR-deficient tumor cells that were appropriately PARP inhibitors sensitive [59]. Guffanti et al. found that in OC-PDX models, higher RAD51 foci levels were correlated with lower sensitivity to PARP inhibitors and lower sensitivity to DDP. Although some studies indicated the potential of RAD51 as a homologous recombinant biomarker, there are still some limitations to its clinical application. Besides, it can be false-negative sometimes [60, 61]. b. CtIP C-terminal binding protein(CtBP) interacting protein (CtIP) physically interacts with the MRE11-RAD50-NBS1 (MRN) complex at DSBs, to promote the shearing of DNA terminal, the formation of ssDNA and activate the MRN complex nuclease in HR repair [62]. Lin et al. found that CtIP inhibitions are related to the sensitivity of PARP inhibitors. It has also been shown to induce the recovery of the replication fork in a FANCD2-dependent manner [63, 64]. Some studies have found that a high CtIP level is a predictor of reduced response to PARP inhibitors and increased response to BRD4i—PARP inhibitors combination therapy [65]. c. FA Fanconi Anemia (FA) is a blood system disease caused by gene changes. The patients’ blood system has aplastic anemia and they also have other congenital malformations. It is mainly due to the instability of the genome and chromosomal abnormalities. Tumor cells with a deficiency in the FA/BRCA pathway are sensitive to PARP inhibitors and other chemotherapies that cause DNA interstrand crosslinks. d.BRCA1 or RAD51C methylated, There is evidence that promoter methylation of BRCA1 and RAD51C genes can reduce the expression of HRR-related genes and results in HRD [66, 67]. Homozygous BRCA1 promoter methylation predicts response to rucaparib in a cohort of 21 ovarian cancer patients in the ARIEL2 trial [68]. Zygosity of BRCA1 methylation was recently identified as a key determinant of PARP inhibitors response using a cohort of HGSC patient-derived xenograft models. Kondrashova et al. demonstrated that all copies of BRCA1 must be methylated to produce a PARPi response and that methylation-loss of a single copy of BRCA1 is sufficient to restore HRR DNA repair and lead to platinum or PARPI resistance [68]. e. ATM Immunohistochemical analyses have been used to test the expression of tumor suppressor proteins. However, because of the low expression of BRCA1/2-related proteins, its use was restricted. In non-BRCA DDR proteins, it has been carried out clinically.

### Potential biomarkers

Sheta et al. used microarray analysis founding that in ascites-derived primary cell cultures (AsPCs)-matched (Highly-grade serous ovarian cancer)HGSOC tumors, C-MET, CDKN2A, N-cadherin and P-glyc/ABCB1 significantly express low levels in PARP inhibitor sensitive AsPCs while SPRY2, E-cadherin and FANCFex express high levels. The 3D functional assay is relatively simple but it still has a certain hint of significance [69].

## Clinical application of PARP inhibitor

### Currently approved

At present, we introduce several PARP inhibitors recommended by 2022 NCCN clinical practice guidelines (Table 1). Olaparib is the first PARP inhibitor listed in the world. In 2018, the US Food and Drug Administration approved olaparib for first-line maintenance treatment in advanced epithelial ovarian patients. These patients must respond fully or partially after platinum-based chemotherapy. The study was based on the SOLO-1 trial, a phase III trial, in which olaparib significantly improved disease progression and prolonged the PFS in patients taking olaparib [70]. Two years later, the FDA approved the combination of olaparib and bevacizumab for first-line maintenance treatment based on the results of PAOLA-1 (nct03737643). It is also approved for patients associated with homologous recombination defect positive status and genomic instability [57].In April 2020, based on the results of the PRIMA trial, FDA approved niraparib for maintenance. In this phase III trial, patients can benefit from niraparib after platinum-based chemotherapy, regardless of whether they have homologous recombination repair defects [71]. Based on the data of ARIEL3, FDA approved the maintenance of rucaparib in recurrent ovarian patients who are platinum-sensitive [72].

**Table 1:** PARP inhibitor recommended by 2022 NCCN clinical practice guidelines

PARP inhibitor	Approved year	Indication and Usage	Recommended dose	Associated clinical trial
Olaparib	2017 by FDA	platinum sensitive, recurrent ovarian cancer for maintenance treatment	300 mg (two 150 mg tablets) taken orally twice daily	SOLO-2 and Study 19
	2018 by FDA	BRCA1/2- mutated advanced ovarian cancer for first-line maintenance treatment	300 mg (two 150 mg tablets) taken orally twice daily	SOLO-1
	2020 by FDA	platinum sensitive and homologous recombinant deficient positive advanced ovarian cancer for combination with bevacizumab for first-line maintenance treatment	300 mg taken orally twice daily	PAOLA-1
Niraparib	2017 by FDA	platinum sensitive and recurrent ovarian cancer for maintenance treatment	based on body weight or platelet count	ENGOT-OV16/NOVA Trial
	2019 by FDA	HRD-positive ovarian cancer for maintenance treatment	300 mg taken once daily	NCT02354586
Rucaparib	2016 by FDA	BRCA1/2-mutated after receiving at least two lines of prior chemotherapy	not mention	Study 10 and ARIEL2
	2018 by FDA	platinum sensitive, recurrent ovarian cancer for maintenance treatment	600 mg (two 300 mg tablets) taken orally twice daily	ARIEL3

**Fig. 3:**
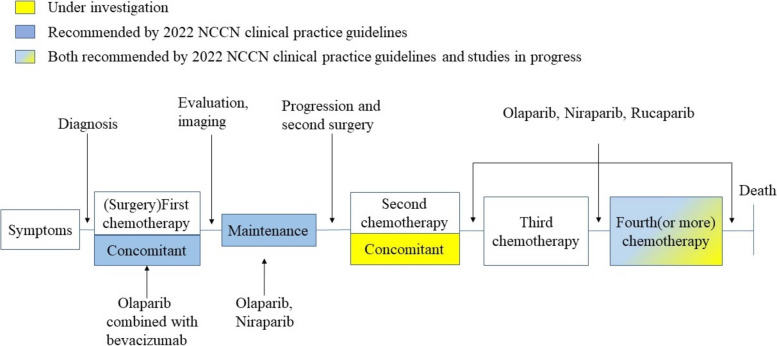
[The indications of PARP inhibitors] In the treatment of ovarian cancer, different colors represent the clinical status of PARP inhibitors in different periods

According to 2022 NCCN clinical practice guidelines to ovarian cancer, PARPi’s maintenance treatment principles and time points are as follows(Fig. 3).

With the popularization and application of PARP inhibitors in the clinic, especially in the treatment of ovarian cancer, the problem of drug resistance has limited its use troubling all oncologists. To overcome its resistance and improve its effect, more and more studies have been carried out in combination with PARP inhibitors. It is worth pointing out that Proteomic techniques, such as mass spectrometry and protein array analysis, promote the analysis of potential molecular signaling events and the study of proteomic characteristics of several cancers. In this context, proteomic analysis of ovarian cancer and its adaptive response to treatment can detect new treatment options, thus reducing the occurrence of drug resistance and potentially improving the prognosis of patients [73].

### Antiangiogenic agents

PARP inhibitors and antiangiogenic agents have shown significant efficacy in the mono treatment of ovarian cancer. In 2020, FDA has approved olaparib with bevacizumab which we have talked about above [74]. It shows the potential of antiangiogenic agents with PARP inhibitors.

Clinically, we observed that most malignant tumors have a richer blood supply than benign tumors. More blood means more nutrition and cancer cells can grow faster and metastasize to other organs. One of the reasons for the abundant blood supply of tumor cells is the increased expression of hypoxia-inducible factor-1(HIF-1). HIF-1 consists of α subunit and β subunit (HIF-1α and HIF-β). HIF-1α is hydroxylated by HIF prolyl-hydroxylase, which then targets HIF-1α for degradation under normoxic conditions. The hydroxylated HIF-1α is specifically ubiquitinated by von Hippel-LindauE3 ubiquitin ligase, which marked the degeneration of HIF-1α proteasome. Under hypoxic conditions, the hydroxylation of HIF-1α is limited by the disposal of oxygen molecules, and HIF-1α is immobilized and assembled. HIF-1α binds to HIF-β and promotes the transcription of hypoxia survival genes. One of the transcripts managed by HIF-1 is vascular endothelial growth factor [75]. Vascular endothelial growth factors (VEGFs A-D) and their receptors (VEGFRs 1–3) can regulate angiogenesis. It has also been found that it is related to lymph node metastasis. Antiangiogenic drugs not only inhibit tumor angiogenesis but also cause hypoxia in the tumor, resulting in DNA damage [76, 77]. It has been found that inhibiting VEGFR3 and blocking the expression of VEGF-C in ovarian cancer can reduce BRCA1 and BRCA2, which is conducive to chemotherapy and the subsequent use of PARP inhibitors [78]. In EVOLVE trial, a phase 2 trial of advanced ovarian patients who were PARP inhibitors resistant, they found patients can benefit obviously from cediranib–olaparib combination treatment for maintenance and it was safer by reducing the dose of cediranib. They even found that the reversion of HR repair-related genes would result in a poor prognosis and the combination treatment was useless for it [79]. In AVANOVA2, the researchers found that the progression-free survival rate of the combination of niraparib and bevacizumab group was significantly improved by about 2 times compared with niraparib alone. In the subgroup of patients without a germline BRCA mutation, niraparib plus bevacizumab also improved progression-free survival compared with niraparib alone [80]. At present, the mechanism of the combined treatment of these two drugs is being studied. Although the combined use of drugs significantly increases efficacy, the aggravation of drug toxicity should also needs further attention.

### ATR/CHK1 inhibitors

ATR/CHK1 pathway helps to stabilize RF. This is mainly because it can prolong the checkpoints of the S phase and G2-M phase in the replication process so that the damage can be repaired in time. Therefore, ATRi can lead to the instability of RF, resulting in apoptosis. CHK1 inhibits cdc25 phosphatase, resulting in cell cycle arrest, which affects the process of DSB damage repair [81]. CAPRI and OLAPCO have found that patients can respond to ATRi—PARPi combination therapy. But these trials have a small number of patients, so the generalization still needs careful consideration. Tumor regression and increased overall survival in patients with acquired PARP inhibitors resistance were observed in patients with BRCA1/2 mutations and BRCA wild-type models [82]. In many preclinical studies, some researchers found that in PARP inhibitors resistant HGSOC cells, the ATR/CHK1 pathway was more active. It may imply the potential of combination [83].

### Immune checkpoint inhibition

In recent years, immune checkpoint inhibitors including anti-PD-1 antibody, anti-PD-L1 antibody, and so on, are in the upsurge of anticancer drug research, although the effect of monotherapy in EOC is not very obvious and still needs expert consensus. When used in combination with PARP inhibitors, it can activate STING mediated antitumor immune response and improve the effect. The clinical model also proved that PARP inhibition inactivated GSK3 and upregulated PD-L1 in a dose-dependent manner. As such, the activation of T cells is inhibited, resulting in increased apoptosis of cancer cells [84]. In murine ovarian cancer models, the combination of PARP inhibitors and anti-PD-1/ PD-L1antibodies augmented the effect of PARP inhibitors [85]. In TOPACIO trial, a phase I/II trial, showed that in platinum-resistant ovarian cancer, niraparib and pembrolizumab showed potential [86]. Some clinical trials are ongoing.

### BET proteins

Bromodeoxyribonucleic acid and extra terminal (BET) protein family is related to the proliferation and survival of cancer cells. BRD4 amplification was observed in HGSOC patients with poor prognosis [87]. Sun et al. showed that BRD4 inhibitors can reduce PARP inhibitor resistance by reducing CTIP which is related to DNA terminal excision and HR repair [65]. A recent study showed that a kind of BETi, JQ1, worked with PARP inhibitors and the combination was more effective in a xenograft model. Other studies have found that BRDi can improve the response of ovarian cancer cells to PARP inhibitors by down-regulating HR-related factors and cause DNA instability through NHEJ pathway [88, 89]. These provide more options for clinical combination treatments. However, further clinical trials are needed to evaluate the toxicity and tolerance in patients.

### PI3K/AKT/mTOR signalling pathway

The phosphatidylinositol-4,5-bisphosphate 3-kinase (PI3K) / AKT/mTOR signal transduction pathway is a common signal transduction pathway in normal cells. However, this pathway is interfered in tumor cells. So far, the anti-tumor effect of single PI3K/AKT inhibitors is limited. Pre-clinical data show that PARPI and PI3K pathway inhibitors have a synergistic effect on anti-tumor, which is achieved through a variety of mechanisms centered on the induction of HRD phenotype. For example, PI3K inhibitors can down-regulate BRCA1/2 and induce HRD [90], and mTOR inhibitors induce inhibition of DNA double strand break (DSB) repair protein SUV39H1, thus inhibiting the expression of HRR gene so as to improve the anti-tumor effect of PARP inhibitors [91].

### RAS/RAF/MEK pathway

PARPi resistance is related to the upregulation of RAS/MAPK pathway, so intervention of MAPK pathway may be one of the related reasons for PARPi re-sensitization. In vitro and in vivo data show that the combination of MEK and PARP inhibition can induce more DNA damage, and may even induce cell death and enhance PARPi activity, prolong the duration of its action and expand its scope of action [92, 93]. Phase I/II clinical trials of Olaparib and selumetinib (MEK inhibitor, NCT03162627) are underway.

### ALK inhibitor

A recent study reported that anaplastic lymphoma kinase (ALK) can directly phosphorylate tyrosine-19 of CDK9. Phosphorylated CDK9-Tyr19 can increase its kinase activity and nuclear localization, stabilize positive transcription elongation factor b, and activate the transcription of HR repair genes dependent on polymerase II, thus promote homologous recombination (HR) repair and increase tumor resistance to PARP inhibitors. Using human tumor biological samples, they further demonstrated that the expression of phosphorylated ALK (p-ALK) was associated with resistance to PARP inhibitors and positively correlated with the expression of p-Tyr19-CDK9 [94]. It can be concluded that ALK and PARP inhibitors can overcome the drug resistance of tumors to PARP inhibitors to some extent.

### EZH2 inhibitor

Recently, EZH2 inhibitors have aroused a heated discussion. A revolutionary drug design, a combination of PARP inhibitors and EZH2 inhibitors based on PROTAC technology, provides a possible solution to PARP inhibitor resistance. EZH2 interacts with PARP through DNA homologous recombination, DNA replication, post-translational modification and tumor immunity. EZH2 inhibitors have potential sensitizing effect on PARP inhibitors. EZH2 inhibitors up-regulate the permeability of immune cells in tumor microenvironment and induce reprogramming of immunosuppressive cells, thus enhancing the killing effect of PARP inhibitors on tumor cells. However, the combination of these two drugs will also disrupt the immune microenvironment of tumors, and in different tumors and different molecular types of tumors, the combination of the two drugs needs to be further explored [95].

PARP inhibitors combined with other drugs will hopefully overcome their limitations and improve their therapeutic efficacy. In addition to the drugs mentioned above, there are other promising combination treatments for PARP inhibitors. At present, most of the clinical trials on the combination of PARP inhibitors are in phase I or II. The following is a list of some representative ongoing or completed clinical trials on the website http://clinicaltrails.gov (table.2). These experimental results will play an important role in the follow-up study of PARP inhibitors.

**Table 2:** Clinical trials related to the combination therapy of PARP inhibitors

Trail ID	Therapeutic Drugs	Phase	Status	Cancer type	Primary outcomes
NCT04669002	EP0057 Olaparib tablets	II	Recruiting	Ovarian Cancer	Overall Response Rate
NCT01623349	BKM120 and Olaparib BYL719 and Olaparib	I	Completed	Ovarian Cancer breast cancer	Maximum tolerated dose Recommended Phase 2 dose
NCT03462342	Olaparib Pill AZD6738	II	Recruiting	High Grade Serous Carcinoma	Incidence of treatment-emergent adverse events Response rate
NCT05494580	Pamiparib Surufatinib	I and II	Not yet recruiting	Ovarian Cancer Ovarian Carcinoma Platinum-resistant Ovarian Cancer Fallopian Tube Carcinosarcoma Primary Peritoneal Cancer	Maximum tolerated dose Recommended Phase 2 dose Response Rate
NCT05071937	ZEN003694 Talazoparib	II	Recruiting	Ovarian Cancer Peritoneal Cancer Fallopian Tube Cancer	Objective Response
NCT04267939	Elimusertib (BAY1895344) Niraparib	I	Recruiting	Advanced Solid Tumors (Excluding Prostate Cancer) Ovarian Cancer	Maximum tolerated dose Recommended Phase 2 dose
NCT04566952	Anlotinib Olaparib	II	Recruiting	Ovarian Cancer Ovarian and Fallopian Tube Cysts and Neoplasms Neoplasms by Site Neoplasms Genital Neoplasms, Female Urogenital Neoplasms Neoplasms, Glandular and Epithelial Neoplasms by Histologic Type Carcinoma, Ovarian Epithelial Ovarian Diseases Adnexal Diseases Genital Diseases, Female Carcinoma Anlotinib PARP Inhibitors BRCA1 Mutation Angiogenesis Antineoplastic Agents BRCA2 Mutation	Progression Free Survival Adverse events
NCT02681237	Cediranib Olaparib	Not Applicable	Completed	Ovarian Cancer	Objective Response Rate Progression-Free Survival Rate
NCT05295589	Copanlisib Hydrochloride Olaparib Paclitaxel Pegylated Liposomal Doxorubicin Hydrochloride Topotecan Hydrochloride	II	Recruiting	Platinum-Refractory Fallopian Tube Carcinoma Platinum-Refractory Ovarian Carcinoma Platinum-Refractory Primary Peritoneal Carcinoma Recurrent Fallopian Tube Endometrioid Adenocarcinoma Recurrent Fallopian Tube High Grade Serous Adenocarcinoma Recurrent Ovarian Endometrioid Adenocarcinoma Recurrent Ovarian High Grade Serous Adenocarcinoma Recurrent Platinum-Resistant Fallopian Tube Carcinoma Recurrent Platinum-Resistant Ovarian Carcinoma Recurrent Platinum-Resistant Primary Peritoneal Carcinoma Recurrent Primary Peritoneal Endometrioid Adenocarcinoma Recurrent Primary Peritoneal High Grade Serous Adenocarcinoma	Progression free survival
NCT02571725	Olaparib Tremelimumab	I and II	Active, not recruiting	Ovarian Cancer Fallopian Tube Cancer Peritoneal Neoplasms	Recommended Phase 2 Dose Objective response rate
NCT05327010	BET Bromodomain Inhibitor ZEN-3694 Talazoparib	II	Recruiting	Advanced Malignant Solid Neoplasm Advanced Ovarian Carcinoma Metastatic Malignant Solid Neoplasm Metastatic Ovarian Carcinoma Recurrent Malignant Solid Neoplasm Refractory Malignant Solid Neoplasm Stage III Ovarian Cancer AJCC v8 Stage IV Ovarian Cancer AJCC v8	Objective response rate
NCT04149145	M4344 + Niraparib	I	Not yet recruiting	Ovarian Cancer Recurrent	Percentage of patients with treatment emergent adverse events Maximum tolerated dose
NCT02446600	Carboplatin Cediranib Maleate Gemcitabine Gemcitabine Hydrochloride Olaparib Paclitaxel Pegylated Liposomal Doxorubicin Hydrochloride	III	Active, not recruiting	Fallopian Tube Clear Cell Adenocarcinoma Fallopian Tube Transitional Cell Carcinoma Fallopian Tube Undifferentiated Carcinoma Ovarian Clear Cell Adenocarcinoma Ovarian Endometrioid Tumor Ovarian Seromucinous Carcinoma Ovarian Serous Tumor Ovarian Transitional Cell Carcinoma Ovarian Undifferentiated Carcinoma Recurrent Fallopian Tube Carcinoma Recurrent Ovarian Carcinoma Recurrent Ovarian Endometrioid Adenocarcinoma Recurrent Primary Peritoneal Carcinoma	Progression free survival
NCT03330405	Avelumab Phase 1b Talazoparib Phase 1b Avelumab Phase 2 Talazoparib Phase 2	II	Active, not recruiting	Locally Advanced (Primary or Recurrent) or Metastatic Solid Tumors	Dose Limiting Toxicity Overall Response
NCT02484404	Olaparib Cediranib Durvalumab	I and II	Recruiting	Colorectal Neoplasms Breast Neoplasms	Overall response rate Recommended phase II dose

## Adverse effect

Hematological toxicity is one of the most common adverse reactions in PARP inhibitors, and it is easy to appear as early as the first month or two of the treatment process. Surveys have shown that hematological adverse events are the most common causes of grade 3 and 4 events in patients treated with niraparib, and the most common cause of dose adjustment or interruption of treatment [56]. Among them, anemia is the most common. In three phase 3 maintenance trials, all-grade anemia occurred in 85 (44%) of 195 patients treated with olaparib [56, 72, 96]. Therefore, the blood routine examination of patients using PARP inhibitors should be monitored regularly in the clinic. Patients with hemoglobin less than 8 g / dl should stop taking drugs, and give symptomatic support treatment such as iron supplement, platelet production and blood transfusion as appropriate.

Common investigational toxicities of using PARP inhibitors include elevated creatinine, liver enzymes and cholesterol. In the SOLO2 trial, 21 (11%) of the 195 patients treated with olaparib had a grade 1 or 2 increase in creatinine (no grades 3 and 4), compared with 1% in the placebo group [96]. Rucaparib led to increased cholesterol of any grade in 40–84% of patients, among them 2–4% of patients had grade 3 or 4 elevation [96, 97]. However, the increase of creatinine and liver enzymes is not significantly related to liver and kidney toxicity. Experts suggest that liver and kidney function should be comprehensively evaluated according to clinical signs, symptoms and radiological examination.

Other common adverse reactions of PARP inhibitors include fatigue and gastrointestinal reactions, among which nausea is the most common, therefore, the symptoms should be treated with prokinetics agents and antiemetics, and patients should be educated to reduce their psychological burden. PARP inhibitors rarely affect the neurological, respiratory, musculoskeletal, cutaneous, and cardiovascular systems [98]. PARP inhibitors have been shown to cause teratogenicity, embryo-fetal toxicity and death in animal reproduction studies, and should, therefore, be avoided during pregnancy [98].

## The prospect of PARP inhibitor use in ovarian cancer

In summary, we discuss the concept of synthetic lethality and explain how PARP inhibitor kills cancer cells through synthetic lethality. Then, according to a series of principles of PARP inhibitors, we explain several possible causes of drug resistance to PARP inhibitors, and list several biomarkers that may be used as therapeutic effects and prognosis. Finally, we describe the current situation of clinical application and several important side effects of PARP inhibitors.

Recently, On the selection of PARP inhibitors and overcoming drug resistance in patients with ovarian cancer, patient-derived organoids (PDOs) is established as a promising tumor model, it allows functional testing of biomarkers for predicting response to PARP inhibitors in EOC. The research data show that it is a useful model system and can be used to quickly evaluate ovarian cancer with DNA deficiency. Combined with next-generation sequencing genome analysis and PDOs, it can screen the patients suitable for PARP inhibitors, help clinicians make decisions and prolong the survival time of patients [99, 100]. Previous studies have shown that patients have achieved good results with the drugs recommended by this method [100]. However, PDOs also have some limitations, such as the quality of biopsy, tumor microenvironment and other conditions. Therefore, how to better select PARP inhibitors suitable for patients needs more exploration and research.

In the past decades, the clinical application of PARP inhibitors has become mature. it provides a new choice for the treatment of ovarian cancer and makes the treatment of ovarian cancer more diversified. Although PARP inhibitors have been widely used, there are still some clinical problems to be solved and optimized. According to the clinical trials of PARP inhibitors on ovarian cancer, about 80% of patients with highly serous ovarian cancer do not have BRCA mutations. Therefore, to further explore PARP inhibitors, explore their undiscovered mechanisms, and extend the application of PARP inhibitors to HRD patients will greatly improve the value of PARP inhibitors. In addition, PARP protein has a mechanism other than DNA repair, so the benefits of PARP inhibitors may not be limited to BRCA, even BRCAness-related tumors. Therefore, more researches on the molecular mechanism of PARP inhibitors and more clinical trials on the wide application of PARP inhibitors will be of great significance to expand the field of PARP inhibition therapy, increase the choice of patients, improve the prognosis of patients and promote follow-up clinical research.
